# Retraction: Hacking on decoy-state quantum key distribution system with partial phase randomization

**DOI:** 10.1038/srep46943

**Published:** 2018-02-09

**Authors:** Shi-Hai Sun, Mu-Sheng Jiang, Xiang-Chun Ma, Chun-Yan Li, Lin-Mei Liang

Correction to: Scientific Reports 4: Article number: 4759; 10.1038/srep04759 published online: 04 23 2014; updated: 02 09 2018.

The authors of the study request that this Article be retracted.

The authors identified an error in the Matlab code used to generate Figure 3 and Figure 4 of the Article. Although the formulas described in the Article are correct, the mistake in the code led to incorrect estimation of the final key rate when Eve is present. With the corrected code, the authors find that the existence of Eve will rapidly increase the estimated error rate, therefore, the attack described in the Article is invalid for the decoy-state QKD protocol, even if the phase source is unrandomized.

All authors agree to the retraction of the Article.

